# Clinical utility of the exosome based ExoDx Prostate(*IntelliScore*) EPI test in men presenting for initial Biopsy with a PSA 2–10 ng/mL

**DOI:** 10.1038/s41391-020-0237-z

**Published:** 2020-05-07

**Authors:** Ronald Tutrone, Michael J. Donovan, Phillipp Torkler, Vasisht Tadigotla, Tom McLain, Mikkel Noerholm, Johan Skog, James McKiernan

**Affiliations:** 1 Chesapeake Urology Associates, Baltimore, MD USA; 2 Department of Pathology, Icahn School of Medicine at Mount Sinai, New York, NY USA; 3 Exosome Diagnostics GmbH, a Bio-Techne brand, Martinsried, Germany; 4 Exosome Diagnostics Inc, a Bio-Techne brand, Waltham, MA USA; 5 Department of Urology, Columbia University Medical Center, New York, NY USA

**Keywords:** Cancer screening, Diagnostic markers, Predictive markers

## Abstract

**Background:**

The ExoDx Prostate(*IntelliScore*) (EPI) test is a non-invasive risk assessment tool for detection of high-grade prostate cancer (HGPC) that informs whether to proceed with prostate biopsy. We sought to assess the impact of EPI on the decision to biopsy in a real-world clinical setting.

**Methods:**

We conducted a prospective, randomized, blinded, two-armed clinical utility study that enrolled 1094 patients with 72 urologists from 24 urology practices. Patients were considered for prostate biopsy at enrollment based on standard clinical criteria. All patients had an EPI test; however, patients were randomized into EPI vs. control arms where only the EPI arm received results for their biopsy decision.

**Results:**

In the EPI arm (*N* = 458), 93 patients received negative EPI scores of which 63% were recommended to defer biopsy by the urologist and 74% ultimately deferred. In contrast, 87% of patients with positive EPI scores were recommended to undergo biopsy with a 72% compliance rate to the urologist’s recommendation. This led to detection of 30% more HGPC compared to the control arm, and we estimate that 49% fewer HGPC were missed due to deferrals compared to standard of care (SOC). Overall, 68% of urologists reported that the EPI test influenced their biopsy decision. The primary reason not to comply with EPI results was rising PSA.

**Conclusion:**

To our knowledge this is the first report on a PC biomarker utility study with a blinded control arm. The study demonstrates that the EPI test influences the overall decision to defer or proceed with a biopsy and improves patient stratification.

## Introduction

Prostate cancer (PC) affects one in nine men and is the most commonly diagnosed cancer among men in the United States (US) [1]. As of 2019, PC accounts for 20% of incident cancers and 10% of estimated cancer-related deaths [1]. The introduction of the prostate specific antigen (PSA) biomarker blood test in the late 1980s gave rise to a marked increase in the number of PC cases [2] and increased prevalence [3]. This led to earlier detection of aggressive tumors (screening benefit) and increased detection of indolent tumors (screening risk) [4]. Unfortunately, the PSA test is unreliable with respect to detecting high-grade PC (HGPC), leaving a gap for accurate HGPC diagnoses [5–7]. In 2012, the US Preventive Services Task Force (USPSTF) recommended against PSA screening due to limited benefits of broad PSA screening. In 2017, to avoid missing HGPCs and potentially increasing PC mortality, the USPSTF promoted age-specific shared decision PSA testing for men aged 55–69 years [8].

The ExoDx Prostate*(IntelliScore)* (EPI, Exosome Diagnostics, Waltham, MA, USA) test is a urine exosome gene expression assay that does not require pre-collection digital rectal exam (DRE). The EPI test is included in the 2019 PC Early Detection National Comprehensive Cancer Network guidelines and uses an algorithm independent of clinical features to provide a risk score that discriminates benign/low-grade PC (Grade Group, GG1, ≤15.6) from HGPC (GG2+, >15.6) for men aged ≥50 who are in the PSA “gray zone” (2–10 ng/mL) [9, 10]. EPI scores are correlated to HGPC and the previously prospectively validated 15.6 cut-point is designed to reduce unnecessary biopsies. This is critical given the current sepsis rate and prevalence of antibiotic-resistant bacteria associated with transrectal ultrasound (TRUS) guided prostate biopsies [11].

One aspect of new predictive tests is to understand utility in clinical practice. To address this, Exosome Diagnostics partnered with CareFirst BlueCross/BlueShield of Maryland to initiate a prospective, randomized, blinded, two-armed clinical utility study to determine the impact of the EPI test on the shared biopsy decision process between patients and urologists. This study is unique as other utility studies in this space did not include a blinded control arm.

## Materials and methods

### Trial design and oversight

**Fig. 1:**
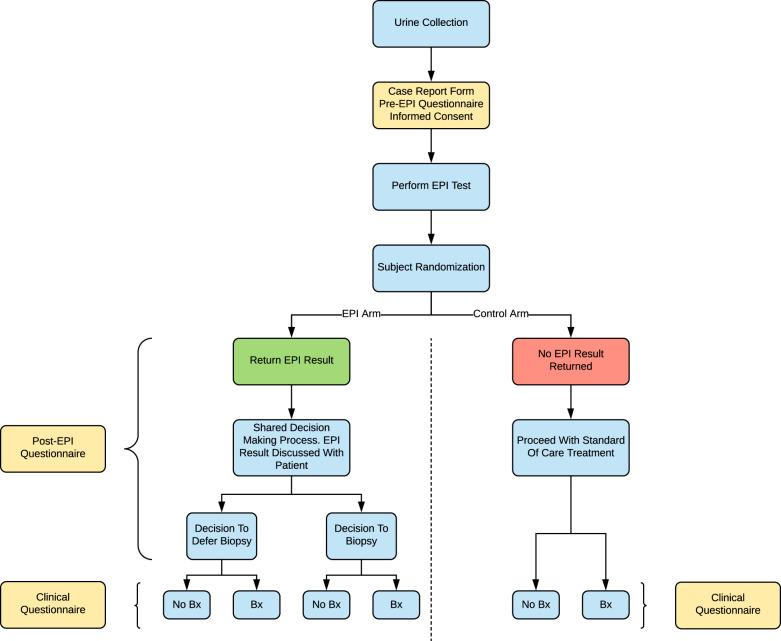
Study flow diagram of the blinded, two-armed clinical utility study of the ExoDx Prostate EPI test. Patients were considered for a prostate biopsy at enrolment based on standard clinical criteria. All subjects had an EPI test; however, patients were randomized into an EPI and control arm where only the EPI arm received the results. Neither subjects nor urologists were notified in advance whether they were going to receive the EPI test result for their clinical assessment. For EPI arm subjects the treating urologist received the test results for review with the patient during the final evaluation prior to biopsy. For control arm subjects, the urologist received a notification of No EPI Result and continued biopsy discussion per standard of care.

After obtaining local institutional review board approvals, we conducted a prospective, blinded, randomized, multi-center clinical utility study from June 2017 to May 2018 (Decision Impact Trial of the ExoDx Prostate(*IntelliScore*), NCT03235687) evaluating shared decision impact of the EPI test among men aged ≥50 with PSA 2–10 ng/mL scheduled for initial 12-core TRUS prostate biopsy in local community clinics. All patients were required to comprehend study documents, provide pre-enrollment written informed consent, and were compensated for their time. Consecutive patients were enrolled, provided a urine sample, and were randomized into two groups: those who would receive the EPI result as part of their biopsy decision process and those who would not (Fig. 1). On enrollment, neither patient nor urologist were notified of inclusion of EPI test results in their post test clinical assessment. EPI arm patients received and reviewed test results with their urologist during the final pre-biopsy evaluation. Control arm patients did not receive their EPI results and continued biopsy discussions per standard of care (SOC). The treating urologist received a questionnaire evaluating the impact of the test in the EPI arm on joint biopsy decisions, utility, ease of understanding, and work-flow implementation. The primary objectives were to see if EPI could reduce initial biopsies by ≥15%, assess how EPI performed on affecting the biopsy decision process at two different risk cut-points (EPI of 15.6 vs 20) and to examine the use of EPI during the biopsy decision process.

### Site identification

Chesapeake Urology Associates, a large Maryland-based community practice, provided all clinical sites. All groups were invited to participate without exception.

### Enrollment criteria

Patients were eligible irrespective of race or ethnicity. Eligible patients were male, aged ≥50 years with clinical indication of PC based on an elevated PSA (2–10 ng/mL) without clinical history of prior negative biopsy. Men with a history of invasive treatment for benign prostatic disease within six months or taking medications affecting PSA levels within 3–6 months were excluded.

### Data collection

The protocol and statistical analyses were developed and agreed upon a priori. Data from each site were collected, managed, submitted according to the study procedures, monitored for timeliness of submission, completeness, and adherence to protocol requirements. Study procedure specifics are in the Supplementary materials including site training, questionnaires, urine transport, electronic data capture protocols, and result reporting.

Patients were assigned de-identified unique identifications, which had been previously randomized between EPI and Control arms irrespective of patient characteristics and site location. The randomization was done according to Kim et al. [12]. Pathology for diagnostic biopsies were performed in conjunction with site-specific pathological services by experienced GU pathologists blinded to EPI test results. The study plan, sample size requirements, objectives, and processes were pre-defined in the clinical trial document (ECT2017–002).

### Assay methods

Details surrounding sample collection, exosome isolation, gene expression, and EPI score generation are in the Supplemental Materials. The EPI cut-point has been previously described and validated [9]. EPI is a risk stratification tool for biopsy decisions. The 15.6 cut-point was selected by a committee of urology key opinion leaders as to what risk would be tolerated to avoid unnecessary biopsies (>90%NPV) without missing clinically significant ≥GG2 PC. The majority of urologists used the recommended cut-point for biopsy deferral in conjunction with SOC to distinguish benign/low-grade PC from HGPC (GG ≥ 2) [9, 13]. Patients with a score <15.6 have a low risk (<9%) of having HGPC on subsequent biopsy, however a urologist may defer a biopsy even with an EPI score above the cut-point when the risk is “low enough”. For example, a patient with an EPI score of 20 has ~20% risk of HGPC, whereas the average patient in the intended use population from the previous validation studies has ~30% risk. The deferral rate due to EPI was captured in the post-EPI questionnaire.

### Statistical analysis

Associations between clinical and demographic factors were evaluated using Student’s *t*-test for continuous variables and Pearson’s Chi-Square test for categorical variables. Shapiro–Wilk’s test was used to compare normally distributed pre-enrollment PSA. The primary endpoint was to determine the percentage of avoided biopsies using the EPI test. The secondary endpoint was successfully diagnosing HGPC. Treatment recommendations (defer or proceed), compliance, risk of HGPC, and final biopsy outcomes are reported as percentages. Data reporting and analyses were conducted using R version 3.6.1 (R Core Team, 2019, Vienna, Austria). Two-tailed *p* values ≤ 0.05 were considered statistically significant.

## Results

**Fig. 2:**
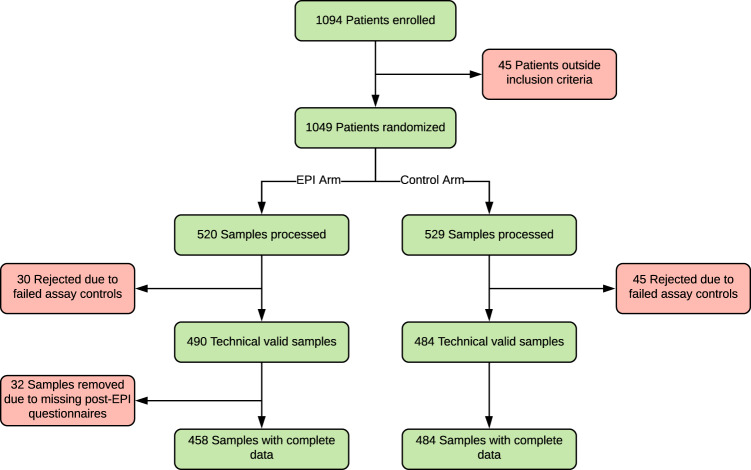
Consort flow diagram. Seventy-two urologists from 24 clinical sites enrolled 1094 subjects to the study. Subjects were excluded if they were outside of the inclusion criteria, failed assay controls or were missing the post-EPI questionnaire, leaving 942 patients for EPI and Control arms.

Twenty-four clinical sites provided data from 1094 patients and 72 urologists. We excluded patients if they were outside the inclusion criteria (*N* = 45, 4.1%), failed assay controls (*N* = 75, 6.8%), or were missing clinical data (*N* = 32, 2.9%). Our final study population was 942 patients (86.1%) with complete data and usable samples (Fig. 2). Among both groups, most patients were Caucasian, with a normal DRE, no PC family history, and median PSA = 4.8 ng/mL. Number, age, demographics, and clinical risk factors were well balanced between the two arms (Table 1). There was a high representation of African-American patients in this study (~23%) and <5% of patients had magnetic resonance imaging (MRI) as part of their SOC assessment (data not shown).

**Table 1:** Demographic characteristics of men considering initial biopsy (*N* = 942).

	EPI arm (*N* = 458)	Control arm (*N* = 484)	*p* value
Demographics	Median (IQR)		
Age (years)	64 (59–69)	65 (59–70)	0.17^*^
PSA (ng/mL)	4.8 (3.9–6.0)	4.8 (3.6–6.2)	0.79^*^
EPI Test Results	28.8 (17.7–46.0)	27.9 (16.2–42.0)	0.10*
	*N* (%)		
PC Family History			
Yes	66 (14.4)	65 (13.4)	0.67^#^
No	387 (84.5)	414 (85.5)	0.66^#^
Unknown	5 (1.1)	5 (1.0)	1.0^#^
Ethnicity			
African-American	102 (22.3)	115 (23.8)	0.63^#^
Asian/Pacific Islander	17 (3.7)	16 (3.3)	1.0^#^
Caucasian	315 (68.8)	320 (66.1)	0.37^#^
Hispanic	9 (2.0)	9 (1.9)	1.0^#^
Other	8 (1.7)	18 (3.7)	0.10^#^
Unknown	7 (1.5)	6 (1.2)	0.92^#^
Digital Rectal Exam			
Normal	406 (88.6)	409 (84.5)	0.08^#^
Abnormal	24 (5.2)	26 (5.4)	1.0^#^
Unavailable	28 (6.1)	49 (10.1)	0.03^#^

Statistical analyses: IQR = Interquartile Range; *t-test; ^*#*^chi-squared test.

*EPI* ExoDx^®^ Prostate(*IntelliScore*), *PSA* serum Prostate Specific Antigen, *PC* prostate cancer.

### Utilization of the EPI test

**Fig. 3:**
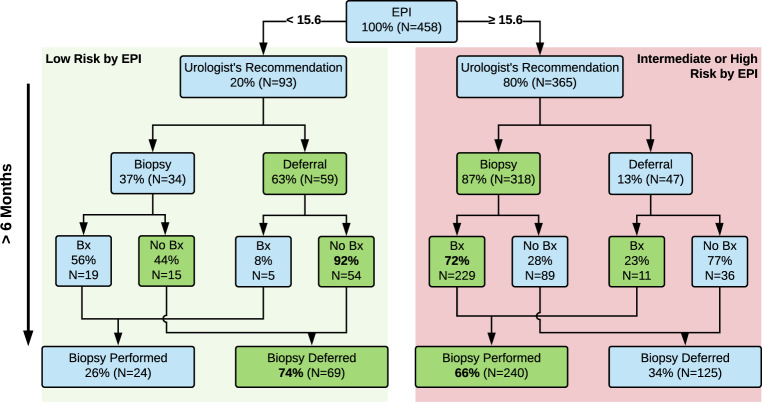
Decision tree and outcome for the EPI arm. Of the 458 subjects that received the EPI test results, 93 were low risk (<15.6) for which 63% were recommended to defer their biopsy with a 92% compliance. The remaining 37% of the EPI low risk subjects were recommended to have a biopsy; however, 44% ultimately decided to defer biopsy. Overall, 69 subjects, representing 74% of all EPI low risk subjects ended up deferring biopsy.

Based on the post-EPI questionnaire, 106 (23.1%) EPI patients were recommended to defer biopsy due to EPI test results (*N* = 59 < 15.6 and *N* = 47 ≥ 15.6), meeting the primary objective of the study (Fig. 3). Ninety-three(20%) patients had an EPI score <15.6; of these, 63% were recommended to defer biopsy and 92% complied (Fig. 3). By comparison, 365 (80%) patients had an EPI score ≥15.6. As anticipated, only a small fraction of these patients (13%) were advised to defer, 87% advised to proceed to biopsy, and 72% complied with the recommendation. Overall, 68% of all urologists felt that the EPI test influenced their decision to biopsy (Supplemental Table S1). Of note, 80% of urologists shared the EPI report with the patient and 98% of urologists and patients found the reports easy to understand. Notably, of urologists who did not change their recommendation post-EPI test, most indicated this was due to presence of a rapidly rising PSA.

### Decision impact of the EPI test

**Fig. 4:**
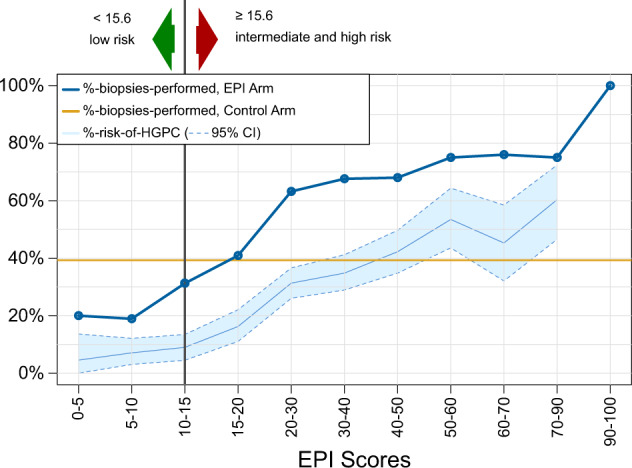
Biopsy decision as a function of EPI risk score. The biopsy decision rate is plotted as a function of the EPI score and superimposed on the light blue area representing risk of HGPC from previous EPI-biopsy outcome validation studies (9,13). Only 26% of EPI low risk (<15.6) subjects proceeded with a biopsy compared to 39% (95% CI: 35–44%) in the control arm (vertical yellow line). As illustrated, urologists used the <15.6 cut-off as a rationale to support biopsy deferral and a >20 EPI score for proceeding with a biopsy due to the increased risk of finding HGPC. An EPI score over 30 indicates an increased risk of finding HGPC relative to patients normally biopsied based on SOC factors (the prevalence of HGPC was 31.6% in the control arm).

The median EPI score in this cohort was 28.8 (Table 1). The performance of EPI was similar to results of our two prior validation studies and are in line with historic data wherein HGPC ( ≥ GG2) baseline risk on biopsy was ~30%, corresponding to an EPI score of ~30 (Fig. 4) [9, 13]. The likelihood of finding ≥GG2 PC increased with increasing EPI score, as shown in Fig. 4. An EPI score of 30 had ~30% chance of finding ≥GG2 PC, and an EPI score of 50 had ~50% chance of finding ≥GG2 PC on 12-core TRUS biopsy. Figure 4 also demonstrates how the EPI test influences biopsy decisions over the entire range of EPI scores. Among patients in the low EPI score range, biopsy rate was reduced, whereas among patients with high EPI scores, biopsy rate increased. Although all enrolled patients considered biopsy due to elevated PSA and/or other SOC clinical parameters, we observed a low biopsy rate in the control arm (39%) vs. the EPI arm (58%). This led to finding 30% more HGPC in the EPI arm compared to SOC. In our study, the EPI test did not miss any patients that were >GG2; it only missed two GG2 patients of the total 264 biopsied patients in the EPI arm.

Retrospective analysis of Chesapeake Urology’s electronic medical records (EMR) from previous years demonstrated similar findings: low biopsy rates (48%) when compared to the study control arm, illustrating the multifactorial nature of biopsy decisions (Supplementary Fig. S1A, B). This further highlights the importance of including a control arm in utility studies. Overall, there was no difference in GG2 disease between the arms; however, the increase in biopsy compliance due to EPI identified 18 additional HGPCs (Supplementary Table S2). We project that the control arm missed 94 HGPC due to high deferral rates, whereas the EPI arm missed 46 (Supplementary Fig. S3). Waterfall plots were used to illustrate biopsy outcomes with EPI scores in both arms (Supplementary Fig. S2). The derived NPV, positive predictive value, sensitivity, and specificity for the control arm was comparable to both previous validation studies (Supplementary Table S3) [9, 13].

### EPI test utility in African-American patients

African-American patients represented 22% and 24% of patients in the EPI and control arms, respectively (Table 1). Of African-Americans, 91% had scores ≥15.6 in the EPI arm, supporting their higher risk of HGPC [14]. The EPI arm found 29 HGPCs whereas the control arm found 16 HGPCs, showing that the increased compliance due to EPI helps identify more HGPC in this high-risk population. The HGPC biopsy ratio increased from 35% in the control arm to 43% in the EPI arm (Supplementary Table S4).

## Discussion

There is increased interest in understanding the role of biomarkers in PC disease, diagnostics, and risk stratification. While many biomarkers have been introduced into clinical practice that can predict outcomes better than SOC [15], how urologists utilize these tools to make management decisions is less clear. EPI is a liquid biopsy urine test that supports personalized risk assessment emphasizing detection of clinically significant ≥GG2 HGPC, and contrary to many other tests in this space, EPI does not include SOC parameters in the risk score. We conducted a prospective, randomized, blinded clinical utility trial with a SOC control arm to understand the impact of EPI scores on the urologist’s decision to proceed or defer biopsy. We found that patients with high EPI scores were more likely to comply with biopsy recommendations, whereas patients with low EPI scores often had their biopsies deferred.

Despite the complexities of the biopsy decision process, these data show that urologists can easily incorporate the EPI test into clinical practice and biopsy the appropriate patient at the right time. Interestingly, all patients in the study were considered for a biopsy; however, ~60% of control arm patients did not follow this recommendation. We determined that this is not unique to this study as post-trial analysis of 2013–2018 Chesapeake Urology historical EMR data identified a similar observed biopsy rate (48%), among other studies with comparable populations [16]. Herein, the majority of patients after one-year did not proceed to biopsy (Supplementary Fig. S2A, B). This highlights the importance of a control arm in biomarker utility studies and elucidates practice patterns that may confound or influence study outcomes.

We also found that given an overall low compliance with urologists’ recommendation for biopsy, detecting HGPC is not compatible with reducing the total number of biopsies performed. While other studies have found that the prevalence of HGPC on TRUS biopsies is ~30%, the actual ≥GG2 prevalence in these patients is substantially higher. The PROMIS study showed the TRUS biopsy sensitivity was 48% and the true prevalence of HGPC based on template prostate mapping biopsies was 53% (95%CI: 49–58%) [17, 18]. Because 12-core TRUS biopsies miss ~50% of ≥GG2 PCs, we estimate ~60% of patients in this cohort have ≥GG2 PC, but only 39% received a biopsy. Given that the percent of patients proceeding to biopsy in the current SOC setting is lower than the prevalence of HGPC in this population, a biomarker test distinguishing HGPC with 100% sensitivity and 100% specificity would not be able to reduce the total number of biopsies among patients being considered for biopsy, but can only help reduce biopsies in test negative patients.

The average risk of finding HGPC upon biopsy in this population is ∼30%, based upon the baseline HGPC detection rate in our control arm as well as both prior validation studies [9, 13]. Patients with EPI scores below the 15.6 cut-point had ~4 times lower risk than the average (∼8%). Detection of HGPC increased with higher EPI scores, and, as seen in Fig. 4, higher EPI scores are associated with higher likelihood of finding ≥GG2 PC on biopsy.

For eligible patients, multiple biomarker assays exist to help improve risk stratification vs. SOC. While these tests have been validated and improve SOC, the degree to which urologists rely on these tests and alter their diagnostic work-up has not been well established. Historically, these other utility studies have not included a blinded control arm. A prospective clinical utility study of 1189 patients with PSA 4–10 ng/mL included the prostate health index (*phi)* in their decision-making compared with a previous historical SOC group from the same large urology group practice that did not receive *phi* [19]. Patients who used *phi* had significantly fewer biopsies. Using *phi* influenced decisions of 73% of cases. However, this study was non-randomized and only used a historical control arm. Another study examined 611 patients who received a 4 K test as part of their pre-biopsy work-up [20]. The 4 K test influenced biopsy decisions in 89% of the patients. Although performing the 4 K test resulted in a 65% reduction in prostate biopsies, this was relative to all patients being recommended to receive a biopsy. Interestingly, the 4 K test does not have a defined cut-point to guide urologists to defer biopsy. As such, the biopsy number may change depending on the individual urologist and their interpretation of the 4 K results. Finally, another study evaluated biopsy rates in patients who received SelectMDx in addition to SOC [21]. Among 418 patients, the authors found that 60% of those with a positive SelectMDx test underwent biopsy vs. only 12% of those with a negative test. However, as data were not captured on the pre-test decision of the urologist, the impact of SelectMDx on a urologist’s biopsy decision process remains unknown. Importantly, because only 40–50% of patients considered for biopsy (first biopsy, PSA 2–10 ng/ml, ≥50 years of age) actually undergo biopsies, reduction in the total number of biopsies would miss a large portion of HGPC.

Indeed, other biomarker tests are NOT likely to reduce biopsy rates unless they either have no correlation to PC or have high rate of missing clinically significant PCs. This misunderstanding is a direct result of their “clinical utility” study design wherein the absence of a blinded control arm compounds the incorrect assumption that all patients in this population receive a biopsy. Furthermore, these biomarkers have claimed to reduce the number of biopsies in utility studies under the assumption that all patients in the targeted population undergo biopsy, which is not the case here or in other studies. None of those studies had a blinded control arm. If EPI used the same incorrect assumption, EPI would reduce biopsies with 27% (fraction of negative patients in the validation study [9].

In the current utility trial, urologists said the EPI test influenced their decision to biopsy in 68% of patients and 23% deferred the biopsy due to the EPI score (Supplementary Table S1). Patients with a low EPI score (<15.6) had 92% compliance with urologist recommendations and the EPI results helped make informed decisions. The EPI test provided complementary information beyond what PSA, family history, ethnicity, or DRE could offer. When a patient/doctor received a high EPI score, they were more likely to comply and perform a biopsy, which led to finding more ≥GG2 clinically significant PC. There are several different metrics that can be applied to a test, finding more PCs or having a higher % ≥GG2 PC among the biopsies. The latter may not necessarily find more PCs. Additionally, the 15.6 cut-point to rule out ≥GG2 PC was not intended to increase the fraction of ≥GG2 PC. For example, if a higher cut-point was used and doctors only biopsied patients with EPI score >30 (30% risk of ≥GG2 PC on biopsy) the % ≥GG2 PC among the biopsies would be much higher (Fig. 4), but may not find as many ≥GG2 PC.

Our study includes racially diverse patients from one of the largest urology group practices in the US. A key strength of our study is that it is a prospective, randomized clinical trial with a blinded control arm. Furthermore, the necessity of a ‘real-world’ control arm as the true comparison, we show that biomarker tests providing both rule out and rule in data elicit complex outcomes highlight that the biopsy decision process is not absolute. Data were collected to examine the relationship between EPI and decisions made by urologists pre/post test. The design was specifically aimed to demonstrate clinical utility, not just correlation between reduced biopsy numbers and the EPI test. Additionally, irrespective of the study arm, enrolled patients were seen by the same urologists and site staff throughout the trial, which allowed for continuity in treatment practices and biopsy decision advisements. Finally, we had a high proportion of African-Americans (23%) relative to the US population (13%). Thus, African-American patients were overrepresented relative to the national average, making this study uniquely generalizable to African-Americans, who are also at higher risk of HGPC on initial biopsy [14]. Using EPI led to detection of more HGPC in African-Americans compared to the control arm. Overall, urologists utilized EPI scores to correctly biopsy this population, supporting the importance of race in the overall decision process.

Despite these strengths, our study does have limitations. There was a 5.7% assay failure rate in the EPI arm (30 assay failures of 520 EPI patients). If we include the patients that were randomized to not receive the EPI test, the failure rate was 7.1%. The failed assay controls is representative of the assay quality control procedures and reflects variations in urine exosome concentration. Although follow-up is ongoing, we currently lack data evaluating long-term outcomes among patients who deferred biopsy after using EPI or any health economics data. We anticipate both aspects will be addressed in the next year. Furthermore, despite our innovative study design, the large number of sites and urologists required streamlined questionnaires, thus limiting comprehensive feedback assessment. We had a small number of patients (<5%) who underwent pre-biopsy MRI. A pre-biopsy MRI has the potential to help refine biopsy accuracy and provide additional information regarding EPI test performance. We also did not use MRI-targeted biopsies in this study as they were not available for us in this real-world clinical setting. Future studies could include a larger percentage of patients with MRI data available.

## Conclusion

This study represents a randomized, prospective clinical utility trial of EPI test for patients undergoing an initial prostate biopsy after abnormal PSA. Our results show that in a real-world clinical setting, EPI influenced the urologist’s behavior with respect to selecting the right patients to biopsy at the right time, thereby improving their ability to identify clinically significant disease and reduce biopsies when the test was negative.

## Supplementary information

Supplemental Material
