# Supplementary material for: Clinical utility of the exosome based ExoDx Prostate(IntelliScore) EPI test in men presenting for initial Biopsy with a PSA 2–10 ng/mL
Source: Prostate Cancer Prostatic Dis. 2020 May 7;23(4):607–14. doi: 10.1038/s41391-020-0237-z (PMC7655505; doi:10.1038/s41391-020-0237-z)
Supplement: Supplementary file 1 — Supplemental Material [file 41391_2020_237_MOESM1_ESM.docx]

**Supplemental Materials:**

*EPI Questionnaires and Biopsy Pathology*

Case report forms, pre/post-EPI test questionnaires, and clinical characteristics such as age (years), PSA (ng/mL), PC family history (yes, no, unknown), were collected at the time of enrollment. After initial consultation and, for subjects randomized to receive the EPI test, review of the EPI result, the subject biopsy status (had biopsy yes/no), and, for subjects who continued with biopsy, the biopsy outcomes (no cancer vs. Gleason grade groups, GG) were recorded. Biopsy pathology was performed by urologic pathologists blinded to the EPI test result at each site. Gleason score (GS) and GG were defined as GS2-6 = GG1, GS 3 + 4 = GG2, GS4 + 3 = GG3, GS8 = GG4 and GS9-10 = GG5 according to the John Hopkins Hospital and International Society of Urological Pathology designations.^12-14^ Subjects are scheduled to be followed for up to five years.

*Assay Methods:*

The EPI test is a non-DRE urine-based liquid biopsy for men aged 50 years or older being considered for initial prostate needle biopsy whose PSA was between 2-10ng/mL. All sites received a urine collection device and shipping kit. All enrolled subjects were instructed to provide 15-20mL of first catch urine samples. Samples were stored at 4-degrees C for up to five days prior to shipping to a centralized processing laboratory (Exosome Diagnostics, Inc., Waltham, MA) for analysis. Description of the methods used in exosome isolation, RNA extraction and reverse transcriptase polymerase chain reaction have been analytically validated and previously published.^9,13^ Results of the EPI test are derived from relative gene expression data of three genes without the inclusion of other clinical parameters. The output of the EPI test is a risk score (scale: 0 – 100), which predicts the presence of high grade PC (>GG2) upon biopsy. EPI cut-points points have been previously described, validated^3^, and shown to discriminate biopsy-positive GG>2 PCs from biopsy GG1 and negative results. Subjects with a score below 15.6 have a low risk (less than 9%) of having high grade PC on subsequent biopsy. The use of the 15.6 cut-point for clinical practice was determined by an external group of urologists from both academia and large urology practice groups as part of the most recent validation study. ^13^The EPI score correlates with risk of HGPC and the 15.6 cut-point was selected to ensure a high NPV (>90%) and the reduction of potential biopsy complications in this population of patients.

*Study Procedure:*

Staff at designated and approved sites received documentation and training to ensure the integrity of the clinical trial. Site staff collected requisition forms, pre/post EPI test questionnaires, instructed subjects on collecting urine samples, stored specimens, shipped specimens and data to Exosome Diagnostics, and interpreted results. Site staff were responsible for identifying and consenting eligible subjects based upon the eligibility criteria described above. All subjects were provided a urine collection device and were required to submit a urine sample at the time of screening and enrollment irrespective of randomization arm. All samples were sent to Exosome Diagnostics for analysis, however, only subjects randomized to the intervention arm had the results of the EPI test presented to them at their next visit.

Site staff, including the treating urologists, were required to fill out and complete the case report form, pre-EPI test questionnaire, and assess baseline clinical characteristics for all subjects on enrollment. In addition to handwritten forms, all sites had access to an electronic data capture software (Medrio e-clinical Systems, San Francisco, CA). These documents were returned to Exosome Diagnostics prior to the final consult meeting with the subject, wherein biopsy planning was discussed. Sites received results of the EPI test within three to five business days after receipt of the samples. Post-EPI test questionnaires were submitted to Exosome Diagnostics after review of the EPI test result between the subject and treating urologist.

Additional clinical information was collected from each site on all enrolled subjects using a clinical questionnaire. Variables collected include whether a biopsy was performed, the results of the biopsy, cancer status (yes/no), cancer outcome (GG/GS), use of antibiotics, pain medicine or any pharmaceutical intervention (additional drugs for lower urinary track symptoms; antibiotic requirements post-biopsy), hospital admission or doctor visits for problems associated with the biopsy, all imaging studies performed and any additional complications as a result of the biopsy.

**Supplemental Table S1.** Impact of EPI Test on combined urologist / patient biopsy decision from the Post-EPI Questionnaire Responses

|  | **Percent of Total Responses** |
| --- | --- |
| 1. Answered yes to: did the EPI test result influence your decision to biopsy this patient? | 67.7 |
| 1. Answered yes to: did you defer the patient’s prostate biopsy based on EPI test results? | 23.1 |
| 1. If results of the EPI test did not change the decision, why?*  - Rapidly Rising PSA - Strong Family History (i.e. Father, Brother) - Race - Nodule/Suspicious DRE - Age - Unanswered | 27.8  6.4  0.9  2.8  11.1  69.9 |

***Multiple choices allowed.**

**Supplemental Figure S1A.** **Chesapeake Urology Electronic Medical Record biopsy rates from 2013-2018 after initial PSA of 2-10ng/mL.** 48% of patients in this population had a biopsy when being followed for up to 5 years.

**Supplemental Figure S1B. Chesapeake Urology Electronic Health Record biopsy rates from 2013-2018 after initial PSA of 2-10ng/mL.** The Likelihood of having an initial biopsy drops significantly after year 1. **63% of the biopsies that are performed happen within the first year and 81% within the first two years. Diminishing number of patients undergo biopsies in subsequent years.**


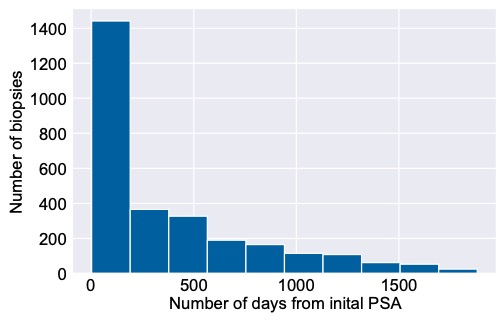


**Supplemental Table S2.** Biopsies Performed and Pathological Findings by Group. The EPI result was only available to EPI arm urologists and patients, but the EPI results from the blinded control arm is also illustrated to illustrate the break-down of EPI below and above the 15.6 cut-point in both arms.

|  | EPI | | | Control | | |
| --- | --- | --- | --- | --- | --- | --- |
|  | **Total** | **EPI < 15.6** | **EPI ≥ 15.6** | **Total** | **EPI < 15.6** | **EPI ≥ 15.6** |
| Biopsy Outcomes | 57.6% (264) | 10% (24) | 90% (240) | 39.3% (190) | 23% (44) | 77% (146) |
| No Cancer | 42.8% (113) | 87.5% (21) | 38.3% (92) | 43.7% (83) | 72.7% (32) | 34.9% (51) |
| GG 1 | 27.7% (73) | 4.2% (1) | 30% (72) | 24.7% (47) | 15.9% (7) | 27.4% (40) |
| GG 2 | 17.4% (46) | 8.3% (2) | 18.3% (44) | 14.7% (28) | 6.8% (3) | 17.1% (25) |
| GG 3 | 8.7% (23) | - | 9.6% (23) | 11.6% (22) | 4.5% (2) | 13.7% (20) |
| GG 4 | 1.9% (5) | - | 2,1% (5) | 1.1% (2) | - | 1.4% (2) |
| GG 5 | 1.5% (4) | - | 1.7% (4) | 4.2% (8) | - | 5.5% (8) |
| $\boldsymbol{\geq}$ GG2 | 29.5% (78) | 8.3% (2) | 31.5% (76) | 31.6% (60) | 11.4% (5) | 37.7% (55) |

**Supplemental Figure S2: Waterfall plots of Patients by EPI Score and Biopsy Outcomes**

A: EPI cohort, B: Control

**Supplemental Table S3.** Classification of performance of EPI in the Control arm, n=190:

|  | % (95% CI) |
| --- | --- |
| % HGPC | 32% (25% - 39%) |
| Predicted negative | 23% (17% - 30%) |
| Sensitivity | 92% (82% - 97%) |
| Specificity | 30% (22% - 39%) |
| NPV | 87% (75% - 96%) |
| PPV | 38% (30% - 46%) |

**Supplemental Figure S3:** Decision tree and outcome distribution of the EPI arm vs the control arm. Due to the high deferral of biopsies that occurs in patients evaluated by SOC (the blinded control arm) it is projected that 94 HGPC will be missed. In contrast, only 46 HGPC is projected to be missed among the deferred EPI arm patients.

**Supplemental Table S4.** Biopsies Performed and Pathological Findings for African-American sub population. The majority of African-American patients received a positive EPI test, leading to more HGPC being detected in the EPI arm.

|  | EPI | | | Control | | |
| --- | --- | --- | --- | --- | --- | --- |
|  | **Total** | **EPI < 15.6** | **EPI ≥ 15.6** | **Total** | **EPI < 15.6** | **EPI ≥ 15.6** |
| Biopsy Outcomes | 67% (68) | 0% (0) | 100% (68) | 40% (46) | 13% (6) | 87% (40) |
| No Cancer | 28% (19) | - | 28% (19) | 26% (12) | 50% (3) | 23% (9) |
| GG 1 | 29% (20) | - | 29% (20) | 39% (18) | 17% (1) | 43% (17) |
| GG 2 | 25% (17) | - | 25% (17) | 13% (6) | 17% (1) | 13% (5) |
| GG 3 | 10% (7) | - | 10% (7) | 15% (7) | 17% (1) | 15% (6) |
| GG 4 | 6% (4) | - | 6% (4) | - | - | - |
| GG 5 | 2% (1) | - | 2% (1) | 7% (3) | - | 8% (3) |
| $\boldsymbol{\geq}$ GG2 | 43% (29) | - | 43% (29) | 35% (16) | 33% (2) | 35% (14) |
